# The dependence of X-ray elastic constants with respect to the penetration depth

**DOI:** 10.1107/S1600576723006878

**Published:** 2023-09-01

**Authors:** Charles Mareau

**Affiliations:** a Arts et Métiers Institute of Technology, 2 boulevard du Ronceray, 49035 Angers, France; Ecole National Supérieure des Mines, Saint-Etienne, France

**Keywords:** X-ray diffraction, X-ray elastic constants, stress analysis, penetration depths, anisotropy, polycrystalline materials, free surfaces

## Abstract

X-ray diffraction techniques are widely used to estimate stresses within polycrystalline materials. The application of these techniques requires the knowledge of the X-ray elastic constants relating the lattice strains to the stress state. Different analytical methods have been proposed to evaluate the X-ray elastic constants from the single-crystal elastic constants. For a given material, such methods provide the bulk X-ray elastic constants but they do not consider the role of free surfaces. However, for many practical applications of X-ray diffraction techniques, the penetration depth of X-rays is the same order of magnitude as the grain size, which means that the influence of the free surface on X-ray elastic constants cannot be excluded. In the present work, a numerical procedure is proposed to evaluate the surface and bulk X-ray elastic constants of polycrystalline materials. While the former correspond to the situation where the penetration is infinitely small in comparison with the grain size, the latter are representative of an infinite penetration depth with no free-surface effect. According to numerical results, the difference between surface and bulk X-ray elastic constants is important for strongly anisotropic crystals. Also, it is possible to propose a relation that allows evaluating X-ray elastic constants as a function of the ratio between the penetration depth and the average grain size. The corresponding parameters of such a relation are provided here for many engineering materials.

## Abstract 2

A numerical method that provides the surface and bulk X-ray elastic constants of non-textured polycrystalline materials is proposed. The results obtained for engineering materials with such a method indicate that, for crystalline materials with significant elastic anisotropy, the X-ray elastic constants depend on the penetration-depth-to-grain-size ratio.

## 1. Introduction

X-ray diffraction techniques (Noyan & Cohen, 1987 ▸; Lu, 1996 ▸) are commonly used for the evaluation of stress states in polycrystalline samples. These techniques rely on the measurement of lattice spacings for one or several lattice planes along different measurement directions. The standard analysis procedure consists of evaluating the corresponding lattice strains. The linear relation between lattice strains and the stress tensor is then exploited to estimate the stress state. Such a relation, which is based on linear elasticity theory, involves some constants that depend on the stiffness properties of the material of interest. As demonstrated by Stickforth (1966 ▸), for non-textured polycrystalline materials, there are two independent constants for each set of equivalent lattice planes. To obtain these constants, which are commonly referred to as the X-ray elastic constants, different strategies have been proposed. Experimental methods consist of imposing a known stress state on a sample of the material of interest, measuring the resulting lattice strains for some lattice planes and estimating the corresponding X-ray elastic constants. While robust and representative results can be obtained with this experimental approach, there are some practical limitations. For example, though only one representative sample of the material is needed, sample preparation is often time consuming. The acquisition of X-ray diffraction data under *in situ* conditions also requires a specific experimental setup to control loading conditions.

As an alternative, some analytical or numerical methods were developed. These methods often rely on homogenization theory to estimate the X-ray elastic constants from single-crystal stiffness constants. For instance, Behnken & Hauk (1986 ▸) used the Voigt (1928 ▸), Reuss (1929 ▸) and Kröner (1958 ▸) approximations to evaluate the X-ray elastic constants of non-textured polycrystalline materials. For textured materials, the macroscopic stiffness properties are anisotropic, and it is generally not possible to describe the relation between lattice strains and the stress tensor with only two constants. However, provided some information regarding crystallographic texture is included, the aforementioned methods can still be used to consider the impact of mechanical stresses on lattice strains (*e.g.* Dölle & Hauk, 1978 ▸, 1979 ▸; Sprauel *et al.*, 1989 ▸). The methods relying on homogenization theory provide the bulk X-ray elastic constants in the sense that they consider each crystal as a domain embedded in an infinite medium. While such an approximation makes sense when the penetration depth is substantial in comparison with the crystallite size, it may not be relevant for the specific, but rather common, situation where the volume probed by X-rays is close to a free surface. In fact, as discussed by van Leeuwen *et al.* (1999 ▸) and Welzel *et al.* (2003 ▸) in the context of thin films, macroscopic stiffness properties may be anisotropic, even in the absence of texture, because of direction-dependent intergranular interactions near free surfaces. Specifically, free surfaces are a possible source of anisotropy because surface grains are not as constrained by their surrounding environment as bulk grains are. As a result, not only does a free surface put some restrictions on the stress tensor, it also affects the dependence of lattice strains with respect to the stress tensor. To consider surface-induced anisotropy, some specific strategies were proposed. For instance, the approach of Vook & Witt (1965 ▸) uses the Reuss approximation for the direction perpendicular to the free surface while the Voigt approximation is adopted for the directions parallel to the free surface. A similar approach was used by Baczmański *et al.* (2003 ▸), who combined the Reuss and Kröner approximations to describe the relation between lattice strains and the stress tensor. These approaches that consider the specific nature of surface grains provide the surface X-ray elastic constants, which correspond to the asymptotic case of a zero penetration depth.

For many applications of X-ray diffraction techniques, the penetration depth is the same order of magnitude as the average grain size. In such a situation, it is difficult to decide whether one should use the surface or the bulk X-ray elastic constants for the evaluation of the stress state. The present work therefore aims at investigating the impact of the penetration-depth-to-average-grain-size ratio on the X-ray elastic constants of non-textured polycrystalline materials. For this purpose, numerical simulations are performed on polycrystalline aggregates to determine the lattice strains for some prescribed loading conditions. The numerical results are then post-processed to evaluate the impact of a free surface on the relation between lattice strains and the stress state. Also, a relation that allows evaluating the X-ray elastic constants for an arbitrary penetration-depth-to-grain-size ratio is proposed. This relation uses the bulk and surface X-ray elastic constants, as well as some additional constants, which are provided for many engineering polycrystalline materials.

## 2. Methods

### 2.1. Surface and volume average quantities

X-ray diffraction techniques provide some information regarding the mechanical and microstructural state of a volume of crystalline material. In the following, the volume probed by X-rays is denoted by 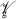 and is referred to as the gauge volume. It is convenient to evaluate some quantities that are spatially averaged over the gauge volume. Specifically, an average quantity (say 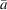 ) obtained from a volume-averaging operation over the gauge volume 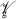 is connected to its local position-dependent counterpart (say *a*) according to

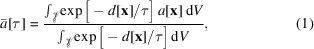

where 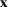 is the position vector, *d* is a function that returns the distance to the free surface and τ is the penetration depth. For the asymptotic case of an infinite penetration depth, the above expression reduces to

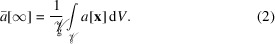

Also, when the penetration depth approaches zero, only the material points lying on the free surface contribute to the average quantity 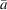 , which means that

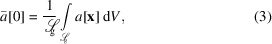

where 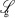 is the free surface illuminated by X-rays.

### 2.2. Definition of X-ray elastic constants

For the evaluation of the stress state, X-ray diffraction techniques rely on the measurement of average lattice strains along different directions within the gauge volume. Specifically, for a given measurement direction defined by the unit vector 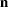 , the gauge-volume average lattice strain 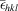 associated with the crystals satisfying diffraction conditions for a set of equivalent lattice planes {*hkl*} is linearly related to the gauge-volume average stress tensor 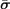 according to

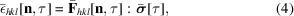

where 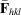 is a second-rank tensor known as the X-ray stress-factor tensor (Hauk, 1997 ▸). Such a tensor depends on the measurement direction, the considered set of equivalent lattice planes, the penetration depth and the microstructure. The role of microstructure is attributed to crystallographic and morphological textures, which both contribute to the anisotropic aspect of macroscopic stiffness properties. Also, the impact of the penetration depth is the result of direction-dependent intergranular interactions near free surfaces.

As demonstrated by Stickforth (1966 ▸), for the specific, but rather common, case of non-textured polycrystalline mat­erials, the bulk X-ray stress-factor tensor, which corresponds to the asymptotic case of an infinite penetration depth, is defined from two constants, denoted by 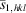 and 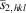 , known as the X-ray elastic constants:^1^


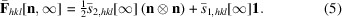

For non-textured polycrystalline materials, equation (4) is thus conveniently written as follows: 

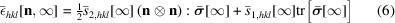

and

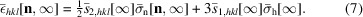

The above relation indicates that, for a set of equivalent lattice planes, the average lattice strain along a specific measurement direction 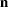 depends on the average hydrostatic stress 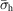 through 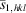 and on the average normal stress 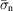 acting along the measurement direction through 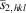 .

For the case of a finite penetration depth, because of surface anisotropy, the above relation is, strictly speaking, not valid. However, one may imagine that, in some situations, the above relation provides a reasonable approximation in the sense that

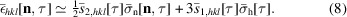

In the following, the impact of the penetration depth on the X-ray elastic constants of non-textured polycrystalline materials is evaluated. For this purpose, two different situations are considered. First, the case of an infinite penetration depth is investigated. Such a situation allows estimating the bulk X-ray elastic constants. In the second situation, different finite penetration depths are considered and the corresponding gauge-volume average X-ray elastic constants are evaluated. The asymptotic case of a zero penetration depth is also considered to determine the surface X-ray elastic constants.

### 2.3. Microstructure generation

**Figure 1:**
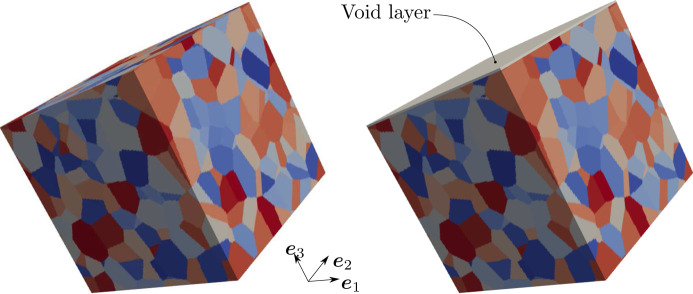
Examples of a bulk microstructure (left) and a surface microstructure (right). Both microstructures consist of 400 crystals represented with random colours. The surface microstructure includes a void layer (grey colour) on the top face.

To evaluate the bulk X-ray elastic constants of polycrystalline materials, a cuboidal periodic volume element (with dimensions *l* × *l* × *l*), such as the one shown in Fig. 1 ▸, is considered. Such a volume element consists of 400 equiaxed grains obtained from a Voronoï tessellation. A triplet of Euler angles is then assigned to each grain according to the procedure of Morawiec (2013 ▸) to obtain a non-textured material. The corresponding microstructure is referred to as the bulk microstructure in the following.

To consider the impact of a free surface on X-ray elastic constants, a similar strategy is adopted to generate the volume element, except that a void layer is added to the top face (see Fig. 1 ▸). The resulting volume element, which represents the surface microstructure, can transmit forces along the directions defined by the 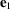 and 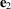 unit vectors, but not along the direction orthogonal to the free surface, which corresponds to the 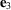 unit vector. For the surface microstructure, both the top and bottom are free surfaces due to the periodicity of the volume element.

Because of the absence of an intrinsic length scale, the penetration depth should be compared with the average grain size. In the present study, the average grain size ϕ is taken as the equivalent spherical diameter: 

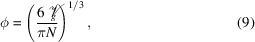

where *N* is the number of grains.

### 2.4. Spectral method

To describe the mechanical behaviour of a given material point, a linear elastic constitutive model is adopted. Specifically, for a material point with position 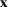 , the local stress tensor 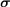 is related to the local infinitesimal strain tensor 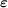 according to

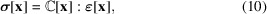

where 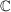 is the fourth-rank stiffness tensor. For the specific case of the void layer, the corresponding material points have zero stiffness properties. The stiffness tensor therefore vanishes for these material points.

In the present work, the spectral method (Moulinec & Suquet, 1998 ▸), which is briefly detailed here, is adopted to solve the field equations resulting from compatibility and equilibrium equations. For a prescribed macroscopic strain tensor 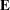 , the spectral method aims at determining the local strain and stress fields that satisfy compatibility conditions, static equilibrium conditions and constitutive equations. In the context of linear elasticity, the local strain field is the solution to the periodic Lippman–Schwinger equation such that

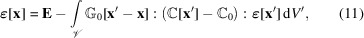

where 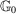 is the symmetric Green tensor, 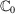 is a reference stiffness tensor and 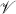 is the total volume. Different iterative procedures (Moulinec & Suquet, 1998 ▸; Eyre & Milton, 1999 ▸; Michel *et al.*, 2001 ▸), which rely on the Fourier transform, have been proposed to solve the above integral equation. In the present work, the original procedure of Moulinec & Suquet (1998 ▸) is used. Also, for the application of the spectral method, a closed-form expression of the symmetric Green tensor in the frequency domain is needed. The expression obtained by Willot (2015 ▸), which results from the application of a centred difference scheme, is adopted for the numerical implementation of the spectral method.

After convergence of the iterative procedure, the macroscopic stress state 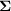 is deduced from the local stress field 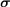 according to the classical averaging relation of homogenization theory:

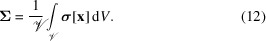

In the general case, the macroscopic stress tensor 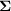 is different from the gauge-volume average stress tensor 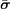 because the gauge volume does not coincide with the total volume of the periodic element. These two stress tensors are equivalent only for an infinite penetration depth (*i.e.* 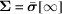 ).

For the application of the spectral method, the polycrystalline volume element corresponding to the bulk microstructure is discretized into 192 × 192 × 192 voxels. For the surface microstructure, two additional layers of voxels are included to represent the void layer. The corresponding volume element is thus discretized into 192 × 192 × 194 voxels.

### 2.5. Numerical evaluation of X-ray elastic constants

For a given set of equivalent planes and a given material, the evaluation of the X-ray elastic constants relies on the post-processing of numerical data obtained from the application of the spectral method. Specifically, the spectral method provides the stress and strain fields resulting from the application of a macroscopic strain and/or stress state to the volume element. These numerical results are then used to compute the gauge-volume average stress tensor and the lattice strains. As detailed hereafter, the gauge-volume average X-ray elastic constants are finally estimated by observing the dependence of lattice strains with respect to the gauge-volume average stress tensor using weighted linear regression analysis.

**Figure 2:**
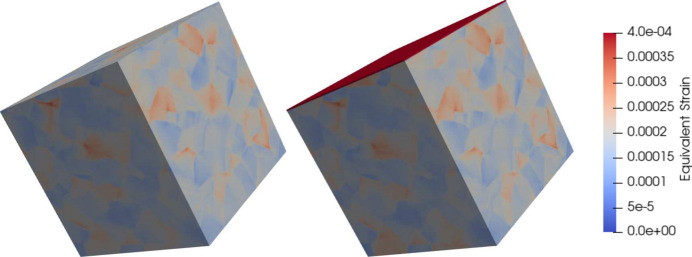
Examples of equivalent strain fields obtained for the bulk microstructure (left) and the surface microstructure (right) of polycrystalline copper. The periodic boundary conditions correspond to the application of a biaxial stress state with an axial strain of 10^−4^. The equivalent strain is given by the Frobenius norm of the strain tensor.

In the following, a biaxial tension state (along 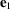 and 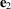 ) is prescribed to the periodic volume element to evaluate the X-ray elastic constants. The prescribed macroscopic strain and stress tensors therefore take the following form:

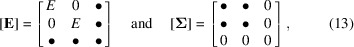

where *E* = 10^−4^ is the prescribed in-plane strain, and the symbol ‘•’ denotes the components of the strain and stress tensors that are adjusted to fulfil boundary conditions. Provided that the gauge-volume average stress tensor contains non-zero spherical and deviatoric parts, the choice of the prescribed macroscopic strain state has no impact on the numerical evaluation of X-ray elastic constants. For the purpose of illustration, the strain fields obtained for the bulk and surface microstructures of polycrystalline copper are displayed in Fig. 2 ▸.

Also, for any material point, the local lattice strain associated with an (*hkl*) lattice plane with unit normal 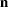 is obtained from

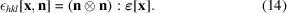

As mentioned earlier, the gauge-volume average X-ray elastic constants are estimated from local lattice strains using weighted linear regression analysis. Specifically, for the application of the weighted least-squares method, the local lattice strains ε_*hkl*_ are the dependent variables, the gauge-volume average normal stresses 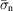 are the independent variables (the total number of independent or dependent variables is given by the product between the number of voxels and the lattice-plane multiplicity), and the average X-ray elastic constants ( 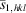 and 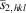 ) appear in the unknown parameters. Also, to consider the impact of the penetration depth, each lattice-strain value is assigned a weight *w* that depends on both the penetration depth and the distance to the free surface: 

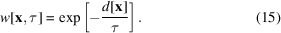

For the evaluation of the bulk X-ray elastic constants (*i.e.* τ = ∞), the weight is equal to unity for any material point. On the other hand, for a finite penetration depth, the weight associated with a given voxel depends on the distance to the nearest free surface. For a set of equivalent planes, the weighted least-squares method thus aims at minimizing the objective function *S*_*hkl*_ such that

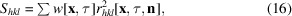

where the residual *r*_*hkl*_ is computed according to 

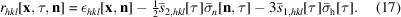




## 3. Results and discussion

### 3.1. The role of surface anisotropy

The role of surface anisotropy is intimately related to the anisotropy of single-crystal stiffness properties. For the specific case of isotropic single-crystal stiffness properties, the X-ray elastic constants do not depend on the penetration depth since the strain field is uniform within the polycrystalline volume element. For such conditions, equations (7) and (8) are therefore equivalent and provide an exact description of the dependence of lattice strains with respect to the stress state. To investigate the role of single-crystal stiffness properties on surface anisotropy, the numerical method described in the previous section[Sec sec2] was used to evaluate the local lattice strains of different fictitious cubic materials. Though general measures of anisotropy can be formulated for any crystal symmetry, the cubic crystal symmetry is examined here because the anisotropic contribution to the stiffness tensor depends on a single scalar parameter. For each material, the lattice strains associated with {200} lattice planes were calculated for both the bulk and surface microstructures. In the former case, local lattice strains were evaluated for all voxels, while only the voxels adjacent to the void layer were considered in the latter case.

For each fictitious cubic material, the single-crystal stiffness properties, given by the three independent components of the stiffness tensor ( 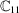 , 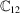 and 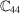 ), were defined from the bulk modulus *K*, the shear modulus *G* and the Zener ratio *Z* according to

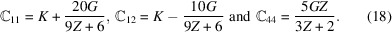

For the different materials, identical values were taken for the bulk and shear moduli (160 and 80 GPa, respectively), but the Zener ratio was changed from unity to infinity. For each material, the corresponding anisotropy index *A* (Kube, 2016 ▸), which, in contrast with the Zener ratio, is not limited to cubic materials, was calculated from the Zener ratio according to

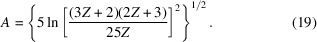

**Figure 3:**
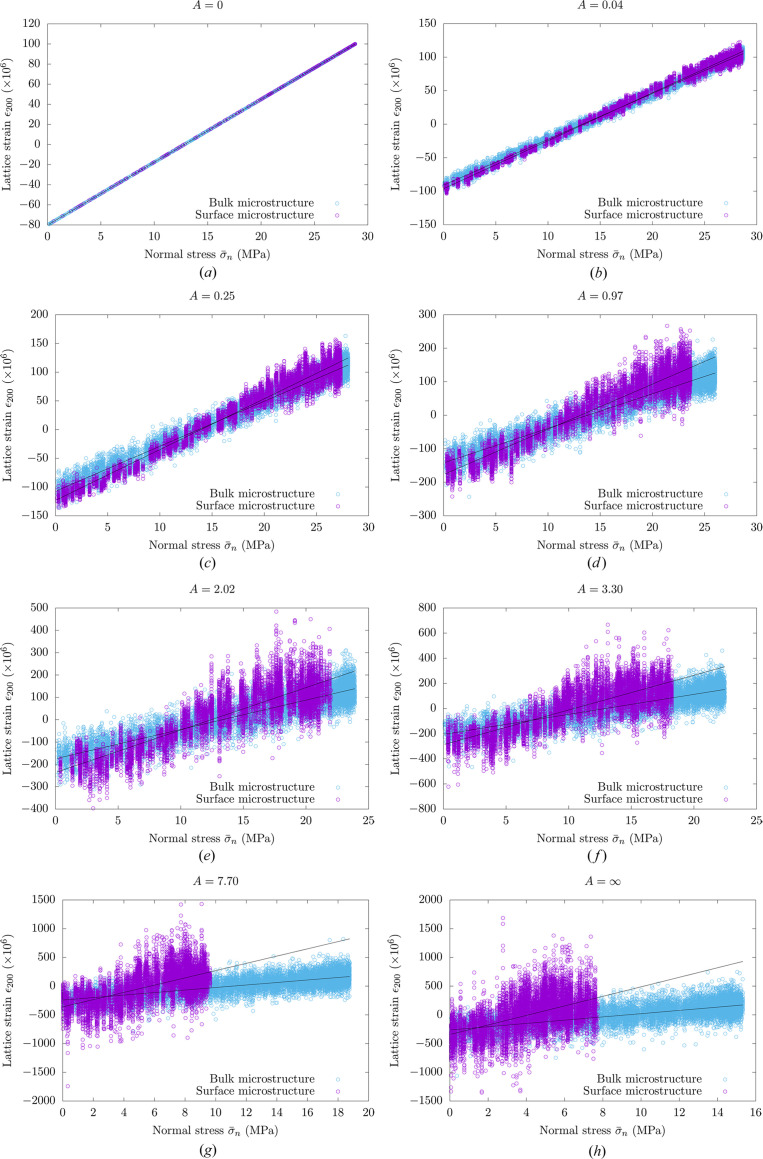
Local lattice strains ε_200_ versus gauge-volume average normal stresses 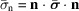 for the {200} planes of cubic polycrystalline materials with different anisotropy indices. Dots correspond to local lattice strains. The results of the weighted linear regression analysis are represented with solid lines. For the bulk microstructure, the local lattice strains were evaluated for all voxels, while only the voxels in contact with free surfaces were considered for the surface microstructure.

The local lattice strains ε_200_ obtained for different anisotropy indices are plotted as a function of the gauge-volume average normal stress 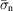 in Fig. 3 ▸. The results of the linear regression analysis are represented with solid lines. For each linear regression, the 95% confidence intervals for the fitting parameters (slope and intercept) associated with the affine approximation were computed. The largest confidence intervals were obtained for the surface microstructure with an infinite anisotropy index. For this specific case, the confidence-interval range represents at most 2% of the fitting-parameter value, which indicates that additional numerical data would not significantly affect the results of the linear regression analysis. For the bulk microstructure with an infinite anisotropy index, the confidence interval is smaller, about 0.1% of the fitting-parameter values.

As discussed earlier, for the specific case of isotropic stiffness properties (*A* = 0), the lattice strains obtained for the bulk and surface microstructures follow the same affine dependence with respect to the average normal stress. When the anisotropy index increases, the free-surface effect becomes clearly visible. In fact, the relation between the local lattice strains and the gauge-volume average normal stress is significantly different for the bulk and surface microstructures. Such results indicate that, for the evaluation of the stress state in materials with strongly anisotropic single-crystal stiffness properties, particular attention should be given to the consideration of the free-surface effect for low penetration depths.

**Figure 4:**
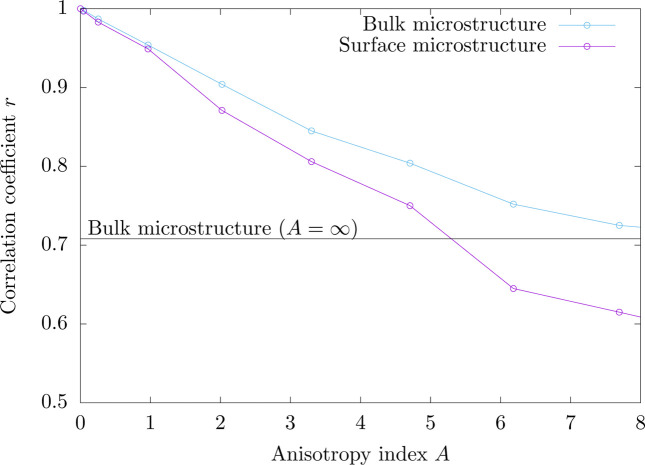
Sample correlation coefficient *r* as a function of the anisotropy index *A* for cubic materials. The sample correlation coefficient was calculated from numerical data (lattice strains and gauge-volume average normal stresses) for bulk and surface microstructures. The horizontal solid line indicates the asymptotic value obtained for the bulk microstructure with an infinite anisotropy index.

For practical applications involving low penetration depths, it is worth determining whether the affine relation provided by equation (8) is reasonable or not. For this purpose, the weighted sample correlation coefficient *r*, which provides a measure of linear correlation between local lattice strains and average normal stresses, was evaluated for both the bulk and surface microstructures as a function of the anisotropy index *A*. As illustrated by Fig. 4 ▸, when the anisotropy index increases, the correlation coefficient decreases. For bulk microstructures, this decrease is the sole consequence of the internal stress field resulting from the heterogeneous aspect of the polycrystalline microstructure. For surface microstructures, the trend is quite similar to that observed for bulk microstructures. However, whatever the anisotropy index is, the sample correlation coefficient is lower for surface microstructures. In fact, the free-surface effect is responsible for some additional deviations with respect to the affine response. If a minimum correlation coefficient of 0.71, which corresponds to the maximum value observed for bulk microstructures, is selected, the affine approximation given by equation (8) provides a reasonable description of the effect of the stress state on lattice strains as long as the anisotropy index remains less than 5.4. As shown in the supporting information, the anisotropy index of common engineering materials is lower than 1, which suggests that the affine approximation is correct for the analysis of the stress state in non-textured polycrystalline materials with low penetration depths.

An alternative way of observing the effect of surface anisotropy consists of computing the out-of-plane stress σ_⊥_ and in-plane stress σ_∥_. These quantities are obtained from the stress tensor according to

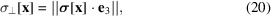




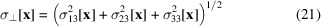

and 

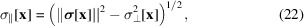




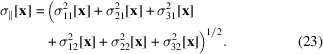

Since 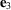 is the unit normal to the free surface, the out-of-plane stress σ_⊥_ must vanish for any material point lying on a free surface while the in-plane stress σ_∥_ may take a non-zero value. To investigate the impact of the distance to the free surface on the stress state, it is convenient to compute the average out-of-plane stress 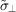 and the average in-plane stress 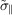 for the surface corresponding to a fixed vertical position *x*_3_. Specifically, they are obtained from

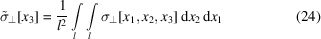

and 

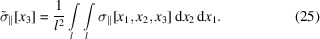

**Figure 5:**
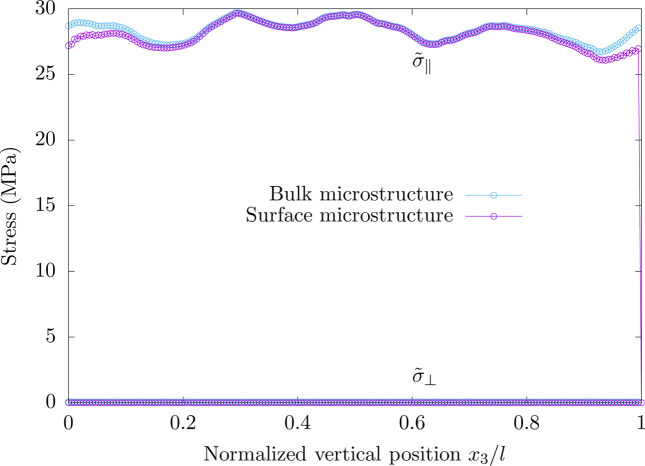
Spatial distributions of the average out-of-plane and in-plane stresses ( 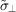 and 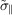 ) obtained for polycrystalline copper. The periodic boundary conditions correspond to the application of a biaxial stress state with an axial strain of 10^−4^. For the surface microstructure, the free surfaces correspond to the positions *x*_3_/*l*_3_ = 0 and *x*_3_/*l*_3_ = 1.

The average out-of-plane and in-plane stresses ( 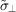 and 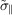 ) obtained for polycrystalline copper are plotted as a function of the dimensionless position *x*_3_/*l* in Fig. 5 ▸. The free surfaces, which are the top and bottom faces of the surface microstructure, correspond to the positions *x*_3_/*l* = 0 and *x*_3_/*l* = 1. According to the results, though the local stress state can be triaxial, the average out-of-plane stress 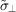 vanishes whatever the distance to the free surface is for both microstructures as a result of mechanical equilibrium. For the average in-plane stress 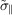 , while the results obtained for the bulk and surface microstructures are similar to each other far from the free surface, the in-plane stress obtained for the surface microstructure is lower than that of the bulk microstructure in the vicinity of a free surface. Such results illustrate the fact that surface grains are less constrained by their surrounding environment than bulk grains. The effect of a free surface on the stress state is visible over a distance that is the same order of magnitude as the grain size ϕ (ϕ/*l* ≃ 0.17 in the present case).

### 3.2. Impact of the penetration-depth-to-average-grain-size ratio

To evaluate the impact of the penetration depth on the X-ray elastic constants of a given material, ten bulk and ten surface microstructures were generated. For each microstructure, the spectral method was then used to solve the field equations. The numerical results were finally post-processed for each individual bulk or surface microstructure to estimate the gauge-volume average X-ray elastic constants for different penetration-depth-to-grain-size ratios (τ/ϕ) ranging from one-tenth to infinity. The values of the gauge-volume average X-ray elastic constants reported in the following are an average of the individual values obtained for the ten different microstructures. The number of microstructures was selected to ensure that, if additional microstructures are considered, the relative effect on average X-ray elastic constants is less than 1%.

**Figure 6:**
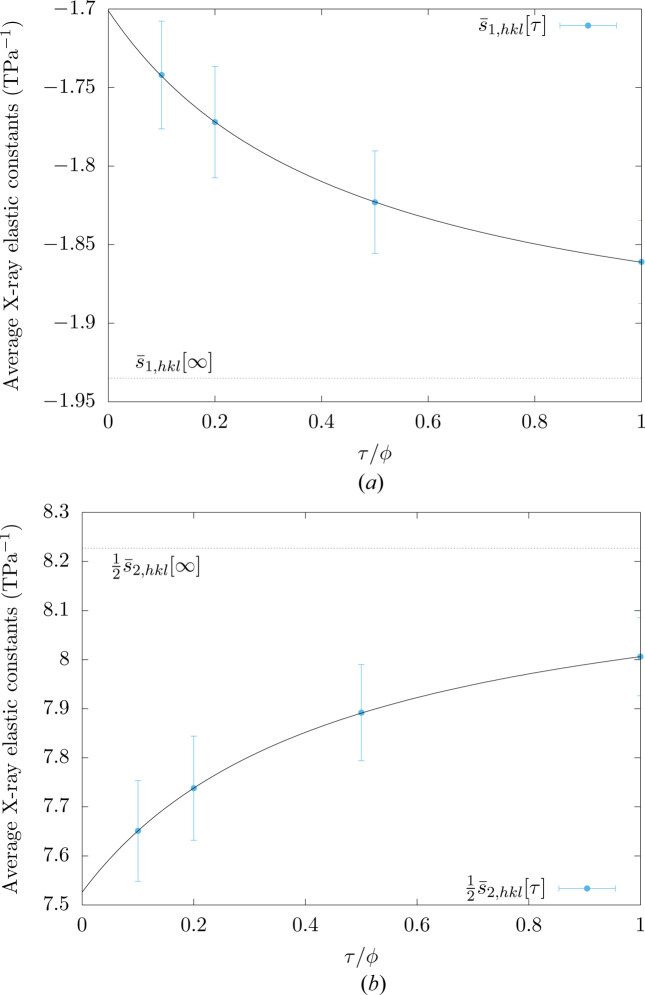
Gauge-volume average X-ray elastic constants ( 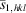 and 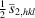 ) for the {111} planes of polycrystalline copper for different penetration-depth-to-average-grain-size ratios. Dots correspond to the average data obtained from numerical results for ten different microstructures. The standard deviations are indicated with vertical error bars. The results obtained from equations (26) and (27) are presented with solid lines. The values of bulk X-ray elastic constants, which correspond to an infinite penetration depth, are indicated with dotted lines.

As discussed in Section 3.1, the X-ray elastic constants depend on the penetration depth. From an engineering perspective, it is useful to have a relation that provides the values of 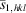 and 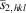 as a function of the ratio between the penetration depth τ and the average grain size ϕ. Such a relation allows determining the X-ray elastic constants for a given penetration depth and a given average grain size. As illustrated in Fig. 6 ▸, the impact of the penetration depth on gauge-volume average X-ray elastic constants is correctly described with the following relations: 

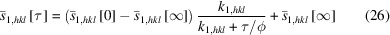

and 

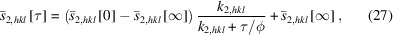

where 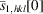 and 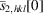 (or 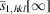 and 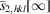 ) are the surface (or bulk) X-ray elastic constants. The former correspond to a zero penetration depth while the latter are associated with an infinite penetration depth. Also, *k*_1,*hkl*_ and *k*_2,*hkl*_ are positive constants that allow considering the impact of the penetration-depth-to-grain-size ratio on the X-ray elastic constants.

**Figure 7:**
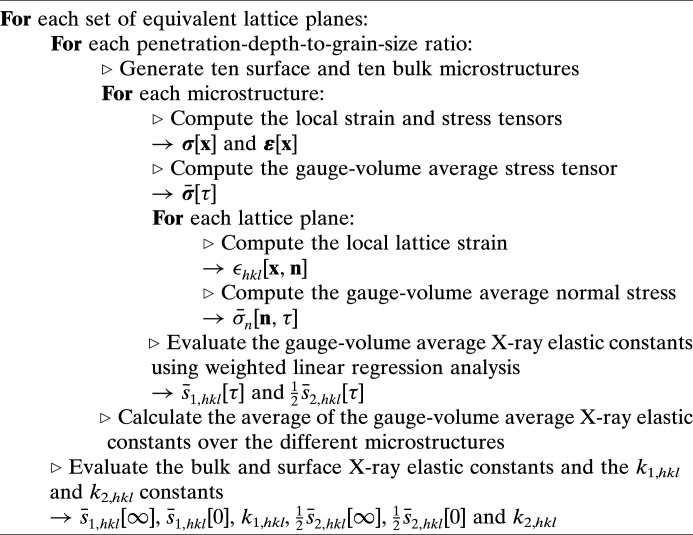
Procedure to obtain surface and bulk X-ray elastic constants, as well as the *k*_1,*hkl*_ and *k*_2,*hkl*_ constants, for different sets of equivalent lattice planes of a given material.

For a given material and given set of lattice planes, the bulk X-ray elastic constants are directly estimated from the numerical results obtained for bulk microstructures with an infinite penetration depth. The evaluation of surface X-ray elastic constants, as well as the *k*_1,*hkl*_ and *k*_2,*hkl*_ constants, uses the numerical results for surface microstructures with different finite penetration-depth-to-grain-size ratios (1/10, 1/5, 1/2 and 1). Specifically, using linear regression analysis, the above constants were adjusted to minimize the difference between the observed gauge-volume average elastic constants and those given by equations (26) and (27). This procedure, which is summarized in Fig. 7 ▸, was used to obtain the surface and bulk X-ray elastic constants, as well as the *k*_1,*hkl*_ and *k*_2,*hkl*_ constants, of different engineering materials (the procedure is explained in more detail in Appendix *A* [App appa]). The corresponding numerical data are presented in the supporting information.

As discussed by Hauk (1997 ▸), the dependence of bulk X-ray elastic constants on lattice planes can be described with some orientation parameters whose definitions depend on the crystal system of the material of interest. For cubic materials, the X-ray elastic constants exhibit an affine dependence with respect to the 3Γ orientation parameter, where Γ is defined according to

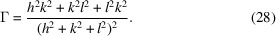

**Figure 8:**
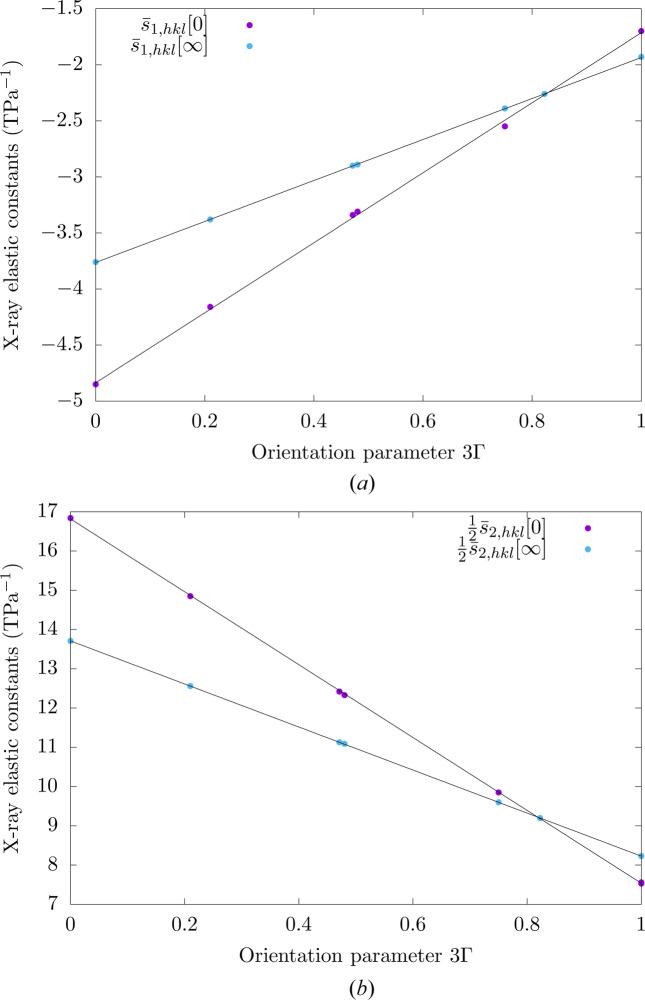
Surface and bulk X-ray elastic constants of polycrystalline copper for different lattice planes as a function of the 3Γ orientation parameter.

As illustrated in Fig. 8 ▸, which shows the X-ray elastic constants obtained for copper as a function of the 3Γ orientation parameter, such an affine dependence is observed not only for the bulk X-ray elastic constants but also for the surface X-ray elastic constants. These results also indicate that, in comparison with bulk X-ray elastic constants, the surface X-ray elastic constants depend more significantly on lattice planes.

For hexagonal materials, a parabolic dependence of the X-ray elastic constants on the orientation parameter *H* ^2^ is observed. This orientation parameter is calculated from Miller indices according to

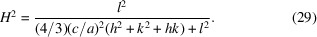

**Figure 9:**
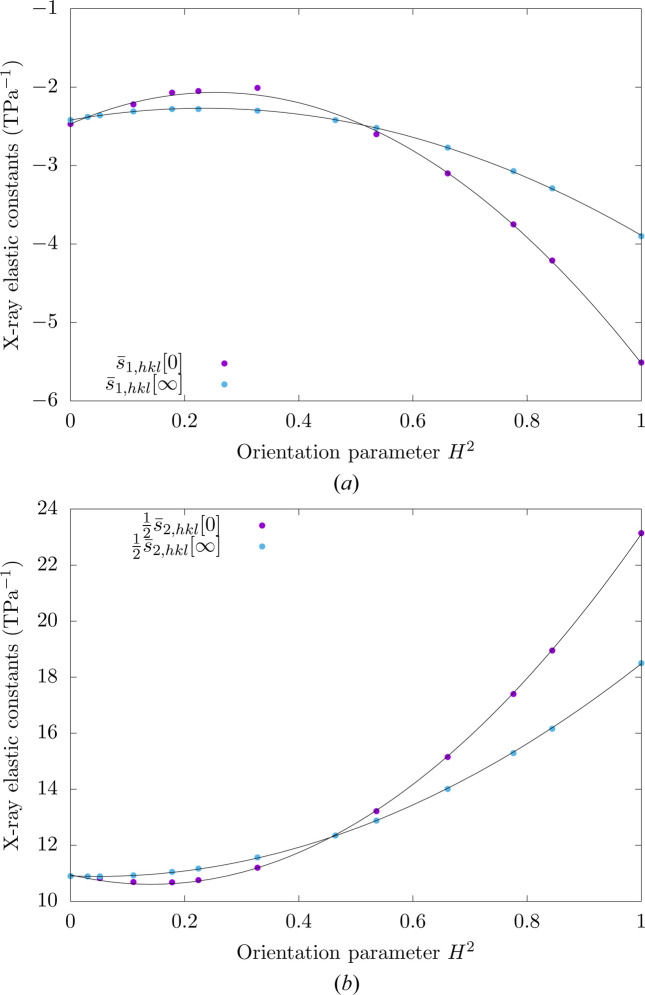
Surface and bulk X-ray elastic constants of polycrystalline zinc for different lattice planes as a function of the *H* ^2^ orientation parameter.

For the purpose of illustration, the X-ray elastic constants obtained for zinc are plotted as a function of the *H* ^2^ orientation parameter in Fig. 9 ▸. The parabolic dependence is observed for both bulk and surface X-ray elastic constants. Also, as for cubic materials, the effect of elastic anisotropy, which controls the dependence of the X-ray elastic constants with respect to the lattice planes, is more pronounced for surface X-ray elastic constants.

In the context of stress analysis, the above results indicate that there are some lattice planes for which the X-ray elastic constants are not much affected by surface anisotropy. These planes should be preferred for stress analysis with X-ray diffraction techniques as the role of the penetration-depth-to-grain-size ratio does not need to be considered to obtain accurate estimates of the stress state. On the other hand, when experimental conditions do not allow one to select such lattice planes, the penetration-depth-to-grain-size ratio should be carefully evaluated, as any error on the X-ray elastic constants would be directly reflected on stress estimates.

## 4. Conclusions

Accurate estimates of X-ray elastic constants are of prime importance for stress analysis in polycrystalline materials with X-ray diffraction techniques. In the present work, the impact of the penetration depth on the X-ray elastic constants of non-textured polycrystalline materials was investigated with a numerical approach. The underlying idea consists of determining whether the X-ray elastic constants are significantly impacted by free-surface effects or not. For this purpose, a numerical approach was proposed to estimate the X-ray elastic constants of polycrystalline materials for different penetration-depth-to-grain-size ratios. This approach relies on the spectral method to determine the stress and strain fields resulting from the application of a macroscopic strain state to a polycrystalline volume element. These numerical results are then used to compute the gauge-volume average stress tensor and the lattice strains. Weighted linear regression analysis is finally applied to estimate the gauge-volume average X-ray elastic constants for a given penetration-depth-to-grain-size ratio. According to the results, the effect of the penetration depth is related to the anisotropic nature of the stiffness properties. In fact, for materials with strongly anisotropic properties, the X-ray elastic constants may significantly depend on the penetration-depth-to-grain-size ratio.

For a given set of equivalent lattice planes and a given material, the X-ray elastic constants for an arbitrary penetration-depth-to-average-grain-size ratio can be obtained from simple analytical relations. Such relations provide a way of interpolating between the surface and bulk X-ray elastic constants, which correspond to the asymptotic cases of zero and infinite penetration depth, respectively. These constants were evaluated for some common engineering materials. The corresponding data, which are reported in the supporting information of this article, are expected to provide valuable information for stress analysis with X-ray diffraction techniques.

## 5. Related literature

The following references are cited in the supporting information for this article: Epstein & Carlson (1965 ▸), Thomas (1968 ▸), Teklu *et al.* (2004 ▸), Leese & Lord Jr (1968 ▸), Soga (1966 ▸), Sumer & Smith (1963 ▸), Wang *et al.* (2019 ▸), Fisher & Renken (1964 ▸), Wazzan & Robinson (1967 ▸), McSkimin & Andreatch Jr (1964 ▸), Alers & Neighbours (1958 ▸), Bogardus (1965 ▸), Deligoz *et al.* (2006 ▸), Gilman & Roberts (1961 ▸), Pizzagalli (2021 ▸), Lee & Gilmore (1982 ▸), Vogelgesang *et al.* (2000 ▸), Fodil *et al.* (2014 ▸), Tefft (1966 ▸), Kandil *et al.* (1984 ▸) and Kisi & Howard (1998 ▸).

## Supplementary Material

Supporting information file. DOI: 10.1107/S1600576723006878/nb5357sup1.pdf

## Footnotes

1 An alternative parametrization consists of using Poisson’s ratio  and Young’s modulus  . These constants are related to  and  with   and  .

## Appendix A Bulk and surface X-ray elastic constants

The surface and bulk X-ray elastic constants, as well as the *k*_1,*hkl*_ and *k*_2,*hkl*_ constants, computed for different engineering materials and different sets of lattice planes are listed in Tables S1–S26 of the supporting information). To obtain these data, the numerical procedure presented in Section 2.5 was applied to evaluate the gauge-volume average X-ray elastic constants for different penetration-depth-to-grain-size ratios (from one-tenth to infinity). The bulk and surface X-ray elastic constants, as well as the *k*_1,*hkl*_ and *k*_2,*hkl*_ constants, were then deduced from the gauge-volume average X-ray elastic constants. For the specific case of similar surface and bulk X-ray elastic constants, which corresponds to the absence of the penetration-depth effect, the *k*_1,*hkl*_ and *k*_2,*hkl*_ constants are not provided as they do not affect the gauge-volume average elastic constants. In the present work, surface and bulk X-ray elastic constants are considered similar when the relative difference is less than 1%.
