# Supplementary material for: The dependence of X-ray elastic constants with respect to the penetration depth
Source: J Appl Crystallogr. 2023 Sep 1;56(Pt 5):1446–55. doi: 10.1107/S1600576723006878 (PMC10543684; doi:10.1107/S1600576723006878)
Supplement: Supplementary file 1 [file j-56-01446-sup1.pdf]

JOURNAL OF APPLIED CRYSTALLOGRAPHY

**Volume 56 (2023)**

**Supporting information for article:**

# The dependence of X-ray elastic constants with respect to the penetration depth

**Charles Mareau**

Table 1: Surface and bulk X-ray elastic constants for copper ($A$ = 0.72 and $a$ = 0.5431 nm). Single crystal elastic constants were taken from Epstein & Clarkson (1965).

| $\{hkl\}$ | $\bar{s}_{1,hkl}\,[0]$ $TPa^{-1}$ | $\bar{s}_{1,hkl}\,[\infty]$ $TPa^{-1}$ | $k_{1,hkl}$ | $\frac{1}{2}\,\bar{s}_{2,hkl}\,[0]$ $TPa^{-1}$ | $\frac{1}{2}\,\bar{s}_{2,hkl}\,[\infty]$ $TPa^{-1}$ | $k_{2,hkl}$ |
|---|---|---|---|---|---|---|
| {111} | -1.70 | -1.93 | 0.46 | 7.53 | 8.23 | 0.46 |
| {200} | -4.85 | -3.76 | 0.38 | 16.84 | 13.71 | 0.38 |
| {220} | -2.55 | -2.39 | 0.06 | 9.85 | 9.60 | 0.06 |
| {311} | -3.34 | -2.90 | 0.33 | 12.42 | 11.13 | 0.33 |
| {222} | -1.70 | -1.93 | 0.46 | 7.56 | 8.23 | 0.46 |
| {400} | -4.85 | -3.76 | 0.38 | 16.84 | 13.71 | 0.38 |
| {331} | -2.26 | -2.26 | – | 9.20 | 9.20 | – |
| {420} | -3.31 | -2.89 | 0.33 | 12.33 | 11.09 | 0.33 |
| {422} | -2.55 | -2.39 | 0.06 | 9.85 | 9.60 | 0.06 |
| {333} | -1.70 | -1.93 | 0.46 | 7.56 | 8.23 | 0.46 |
| {511} | -4.16 | -3.38 | 0.37 | 14.85 | 12.56 | 0.37 |

Table 2: Surface and bulk X-ray elastic constants for aluminium ($A$ = 0.02 and $a$ = 0.40498 nm). Single crystal elastic constants were taken from Thomas (1968).

| $\{hkl\}$ | $\bar{s}_{1,hkl}\,[0]$ $TPa^{-1}$ | $\bar{s}_{1,hkl}\,[\infty]$ $TPa^{-1}$ | $k_{1,hkl}$ | $\frac{1}{2}\,\bar{s}_{2,hkl}\,[0]$ $TPa^{-1}$ | $\frac{1}{2}\,\bar{s}_{2,hkl}\,[\infty]$ $TPa^{-1}$ | $k_{2,hkl}$ |
|---|---|---|---|---|---|---|
| {111} | -4.55 | -4.67 | 0.48 | 18.03 | 18.40 | 0.48 |
| {200} | -5.48 | -5.27 | 0.45 | 20.83 | 20.20 | 0.45 |
| {220} | -4.77 | -4.82 | 0.48 | 18.86 | 18.86 | – |
| {311} | -4.99 | -4.99 | – | 19.36 | 19.36 | – |
| {222} | -4.55 | -4.67 | 0.48 | 18.03 | 18.40 | 0.48 |
| {400} | -5.48 | -5.27 | 0.45 | 20.83 | 20.20 | 0.45 |
| {331} | -4.71 | -4.78 | 0.48 | 18.51 | 18.73 | 0.47 |
| {420} | -4.98 | -4.98 | – | 19.34 | 19.34 | – |
| {422} | -4.77 | -4.82 | 0.48 | 18.86 | 18.86 | – |
| {333} | -4.55 | -4.67 | 0.48 | 18.03 | 18.40 | 0.48 |
| {511} | -5.28 | -5.14 | 0.45 | 20.22 | 19.83 | 0.46 |

Table 3: Surface and bulk X-ray elastic constants for nickel ($A = 0.50$ and $a = 0.35251$ nm). Single crystal elastic constants were taken from Epstein & Clarkson (1965).

| $\{hkl\}$ | $\bar{s}_{1,hkl}\,[0]$ TPa$^{-1}$ | $\bar{s}_{1,hkl}\,[\infty]$ TPa$^{-1}$ | $k_{1,hkl}$ | $\frac{1}{2}\,\bar{s}_{2,hkl}\,[0]$ TPa$^{-1}$ | $\frac{1}{2}\,\bar{s}_{2,hkl}\,[\infty]$ TPa$^{-1}$ | $k_{2,hkl}$ |
|---|---|---|---|---|---|---|
| {111} | -0.89 | -1.02 | 0.39 | 4.45 | 4.84 | 0.39 |
| {200} | -2.36 | -1.91 | 0.44 | 8.87 | 7.52 | 0.44 |
| {220} | -1.24 | -1.24 | – | 5.51 | 5.51 | – |
| {311} | -1.65 | -1.49 | 0.53 | 6.75 | 6.26 | 0.53 |
| {222} | -0.89 | -1.02 | 0.39 | 4.47 | 4.84 | 0.39 |
| {400} | -2.36 | -1.91 | 0.44 | 8.87 | 7.52 | 0.44 |
| {331} | -1.13 | -1.18 | 0.17 | 5.23 | 5.32 | 0.16 |
| {420} | -1.63 | -1.48 | 0.53 | 6.71 | 6.24 | 0.53 |
| {422} | -1.24 | -1.24 | – | 5.51 | 5.51 | – |
| {333} | -0.89 | -1.02 | 0.39 | 4.47 | 4.84 | 0.39 |
| {511} | -2.03 | -1.72 | 0.47 | 7.91 | 6.96 | 0.47 |

Table 4: Surface and bulk X-ray elastic constants for Fe-18Cr-14Ni alloy ($A = 0.75$ and $a = 0.3593$ nm). Single crystal elastic constants were taken from Teklu (2004).

| $\{hkl\}$ | $\bar{s}_{1,hkl}\,[0]$ TPa$^{-1}$ | $\bar{s}_{1,hkl}\,[\infty]$ TPa$^{-1}$ | $k_{1,hkl}$ | $\frac{1}{2}\,\bar{s}_{2,hkl}\,[0]$ TPa$^{-1}$ | $\frac{1}{2}\,\bar{s}_{2,hkl}\,[\infty]$ TPa$^{-1}$ | $k_{2,hkl}$ |
|---|---|---|---|---|---|---|
| {111} | -0.80 | -0.95 | 0.36 | 4.64 | 5.09 | 0.36 |
| {200} | -2.92 | -2.18 | 0.40 | 10.98 | 8.77 | 0.40 |
| {220} | -1.33 | -1.26 | 0.53 | 6.23 | 6.00 | 0.52 |
| {311} | -1.92 | -1.60 | 0.44 | 7.98 | 7.03 | 0.43 |
| {222} | -0.80 | -0.95 | 0.36 | 4.65 | 5.09 | 0.36 |
| {400} | -2.92 | -2.18 | 0.40 | 10.98 | 8.77 | 0.40 |
| {331} | -1.18 | -1.17 | 1.35 | 5.74 | 5.74 | – |
| {420} | -1.90 | -1.59 | 0.44 | 7.92 | 7.00 | 0.44 |
| {422} | -1.33 | -1.26 | 0.53 | 6.23 | 6.00 | 0.52 |
| {333} | -0.80 | -0.95 | 0.36 | 4.65 | 5.09 | 0.36 |
| {511} | -2.41 | -1.92 | 0.51 | 9.63 | 7.99 | 0.51 |

Table 5: Surface and bulk X-ray elastic constants for $\alpha-$iron ($A = 0.51$ and $a = 0.28665$ nm). Single crystal elastic constants were taken from Leese & Lord Jr. (1968).

| $\{hkl\}$ | $\bar{s}_{1,hkl}\,[0]$ $\text{TPa}^{-1}$ | $\bar{s}_{1,hkl}\,[\infty]$ $\text{TPa}^{-1}$ | $k_{1,hkl}$ | $\frac{1}{2}\,\bar{s}_{2,hkl}\,[0]$ $\text{TPa}^{-1}$ | $\frac{1}{2}\,\bar{s}_{2,hkl}\,[\infty]$ $\text{TPa}^{-1}$ | $k_{2,hkl}$ |
|---|---|---|---|---|---|---|
| {110} | -1.33 | -1.31 | 0.43 | 5.92 | 5.92 | – |
| {200} | -2.54 | -2.04 | 0.37 | 9.53 | 8.11 | 0.37 |
| {211} | -1.33 | -1.31 | 0.43 | 5.92 | 5.92 | – |
| {220} | -1.33 | -1.31 | 0.43 | 5.92 | 5.92 | – |
| {222} | -0.94 | -1.07 | 0.38 | 4.83 | 5.18 | 0.38 |
| {310} | -2.10 | -1.78 | 0.38 | 8.26 | 7.32 | 0.38 |
| {222} | -0.94 | -1.07 | 0.38 | 4.83 | 5.18 | 0.38 |
| {321} | -1.33 | -1.31 | 0.43 | 5.92 | 5.92 | – |
| {400} | -2.54 | -2.04 | 0.37 | 9.53 | 8.11 | 0.37 |
| {330} | -1.33 | -1.31 | 0.43 | 5.92 | 5.92 | – |
| {420} | -1.76 | -1.58 | 0.38 | 7.27 | 6.71 | 0.38 |

Table 6: Surface and bulk X-ray elastic constants for $\alpha-$tantalum ($A = 0.11$ and $a = 0.33204$ nm). Single crystal elastic constants were taken from Soga (1966)

| $\{hkl\}$ | $\bar{s}_{1,hkl}\,[0]$ $\text{TPa}^{-1}$ | $\bar{s}_{1,hkl}\,[\infty]$ $\text{TPa}^{-1}$ | $k_{1,hkl}$ | $\frac{1}{2}\,\bar{s}_{2,hkl}\,[0]$ $\text{TPa}^{-1}$ | $\frac{1}{2}\,\bar{s}_{2,hkl}\,[\infty]$ $\text{TPa}^{-1}$ | $k_{2,hkl}$ |
|---|---|---|---|---|---|---|
| {110} | -1.75 | -1.75 | – | 7.00 | 7.00 | – |
| {200} | -2.32 | -2.12 | 0.43 | 8.70 | 8.12 | 0.43 |
| {211} | -1.75 | -1.75 | – | 7.00 | 7.00 | – |
| {220} | -1.75 | -1.75 | – | 7.00 | 7.00 | – |
| {222} | -1.54 | -1.62 | 0.40 | 6.37 | 6.62 | 0.40 |
| {310} | -2.10 | -1.99 | 0.45 | 8.07 | 7.71 | 0.44 |
| {222} | -1.54 | -1.62 | 0.40 | 6.37 | 6.62 | 0.40 |
| {321} | -1.75 | -1.75 | – | 7.00 | 7.00 | – |
| {400} | -2.32 | -2.12 | 0.43 | 8.70 | 8.12 | 0.43 |
| {330} | -1.75 | -1.75 | – | 7.00 | 7.00 | – |
| {420} | -1.94 | -1.88 | 0.46 | 7.58 | 7.40 | 0.46 |

Table 7: Surface and bulk X-ray elastic constants for chromium ($A = 0.07$ and $a = 0.28839$ nm). Single crystal elastic constants were taken from Sumer & Smith (1963).

| $\{hkl\}$ | $\bar{s}_{1,hkl}\,[0]$ $\mathrm{TPa}^{-1}$ | $\bar{s}_{1,hkl}\,[\infty]$ $\mathrm{TPa}^{-1}$ | $k_{1,hkl}$ | $\frac{1}{2}\,\bar{s}_{2,hkl}\,[0]$ $\mathrm{TPa}^{-1}$ | $\frac{1}{2}\,\bar{s}_{2,hkl}\,[\infty]$ $\mathrm{TPa}^{-1}$ | $k_{2,hkl}$ |
|---|---|---|---|---|---|---|
| {110} | -0.80 | -0.77 | 0.44 | 4.59 | 4.50 | 0.44 |
| {200} | -0.52 | -0.58 | 0.38 | 3.76 | 3.93 | 0.38 |
| {211} | -0.80 | -0.77 | 0.44 | 4.59 | 4.50 | 0.44 |
| {220} | -0.80 | -0.77 | 0.44 | 4.59 | 4.50 | 0.44 |
| {222} | -0.89 | -0.84 | 0.40 | 4.87 | 4.70 | 0.40 |
| {310} | -0.62 | -0.65 | 0.34 | 4.06 | 4.13 | 0.35 |
| {222} | -0.89 | -0.84 | 0.40 | 4.87 | 4.70 | 0.40 |
| {321} | -0.80 | -0.77 | 0.44 | 4.59 | 4.50 | 0.44 |
| {400} | -0.52 | -0.58 | 0.38 | 3.76 | 3.93 | 0.38 |
| {330} | -0.80 | -0.77 | 0.44 | 4.59 | 4.50 | 0.44 |
| {420} | -0.70 | -0.70 | – | 4.30 | 4.30 | – |

Table 8: Surface and bulk X-ray elastic constants for the Ti-27Nb-7Al alloy ($A = 0.77$ and $a = 0.3290$ nm). Single crystal elastic constants were taken from Wang *et al.* (2019).

| $\{hkl\}$ | $\bar{s}_{1,hkl}\,[0]$ $\mathrm{TPa}^{-1}$ | $\bar{s}_{1,hkl}\,[\infty]$ $\mathrm{TPa}^{-1}$ | $k_{1,hkl}$ | $\frac{1}{2}\,\bar{s}_{2,hkl}\,[0]$ $\mathrm{TPa}^{-1}$ | $\frac{1}{2}\,\bar{s}_{2,hkl}\,[\infty]$ $\mathrm{TPa}^{-1}$ | $k_{2,hkl}$ |
|---|---|---|---|---|---|---|
| {110} | -5.19 | -4.85 | 0.09 | 18.18 | 17.16 | 0.09 |
| {200} | -9.35 | -7.26 | 0.34 | 30.26 | 24.40 | 0.34 |
| {211} | -5.19 | -4.85 | 0.09 | 17.74 | 17.16 | 0.09 |
| {220} | -5.19 | -4.85 | 0.09 | 17.74 | 17.16 | 0.09 |
| {222} | -3.64 | -4.03 | 0.48 | 13.52 | 14.70 | 0.48 |
| {310} | -7.84 | -6.40 | 0.31 | 25.73 | 21.82 | 0.31 |
| {222} | -3.64 | -4.03 | 0.48 | 13.52 | 14.70 | 0.48 |
| {321} | -5.19 | -4.85 | 0.09 | 17.74 | 17.16 | 0.09 |
| {400} | -9.35 | -7.26 | 0.34 | 30.26 | 24.40 | 0.34 |
| {330} | -5.19 | -4.85 | 0.09 | 17.74 | 17.16 | 0.09 |
| {420} | -6.67 | -5.73 | 0.26 | 22.24 | 19.79 | 0.26 |

Table 9: Surface and bulk X-ray elastic constants for $\alpha-$titanium ($A = 0.08$, $a = 0.29508$ nm and $c = 0.46855$ nm). Single crystal elastic constants were taken from Fisher & Renken (1964).

| $\{hkl\}$ | $\bar{s}_{1,hkl}\,[0]$ TPa$^{-1}$ | $\bar{s}_{1,hkl}\,[\infty]$ TPa$^{-1}$ | $k_{1,hkl}$ | $\frac{1}{2}\,\bar{s}_{2,hkl}\,[0]$ TPa$^{-1}$ | $\frac{1}{2}\,\bar{s}_{2,hkl}\,[\infty]$ TPa$^{-1}$ | $k_{2,hkl}$ |
|---|---|---|---|---|---|---|
| {100} | -3.11 | -2.96 | 0.35 | 12.33 | 11.98 | 0.29 |
| {110} | -3.11 | -2.96 | 0.35 | 12.26 | 11.98 | 0.29 |
| {002} | -2.04 | -2.33 | 0.43 | 9.52 | 10.14 | 0.45 |
| {101} | -2.95 | -2.89 | 0.39 | 11.91 | 11.76 | 0.38 |
| {102} | -2.67 | -2.72 | 0.62 | 11.28 | 11.28 | – |
| {110} | -3.11 | -2.96 | 0.35 | 12.26 | 11.98 | 0.29 |
| {103} | -2.46 | -2.58 | 0.55 | 10.63 | 10.88 | 0.62 |
| {200} | -3.11 | -2.96 | 0.35 | 12.26 | 11.98 | 0.29 |
| {112} | -2.91 | -2.86 | 0.38 | 11.81 | 11.70 | 0.37 |
| {201} | -3.07 | -2.95 | 0.37 | 12.17 | 11.93 | 0.32 |
| {004} | -2.04 | -2.33 | 0.43 | 9.52 | 10.14 | 0.45 |
| {202} | -2.95 | -2.89 | 0.39 | 11.91 | 11.76 | 0.38 |
| {104} | -2.33 | -2.50 | 0.51 | 10.28 | 10.63 | 0.56 |
| {203} | -2.80 | -2.80 | – | 11.53 | 11.53 | – |
| {120} | -3.11 | -2.96 | 0.35 | 12.26 | 11.98 | 0.29 |
| {211} | -3.09 | -2.95 | 0.36 | 12.21 | 11.95 | 0.32 |
| {114} | -2.59 | -2.67 | 0.58 | 11.00 | 11.13 | 0.66 |
| {105} | -2.24 | -2.45 | 0.49 | 10.06 | 10.49 | 0.53 |
| {122} | -3.01 | -2.92 | 0.39 | 12.05 | 11.86 | 0.35 |

Table 10: Surface and bulk X-ray elastic constants for magnesium ($A$ = 0.02, $a$ = 0.32094 nm and $c$ = 0.52108 nm). Single crystal elastic constants were taken from Wazzan & Robinson (1967).

| $\{hkl\}$ | $\bar{s}_{1,hkl}\,[0]$ TPa$^{-1}$ | $\bar{s}_{1,hkl}\,[\infty]$ TPa$^{-1}$ | $k_{1,hkl}$ | $\frac{1}{2}\,\bar{s}_{2,hkl}\,[0]$ TPa$^{-1}$ | $\frac{1}{2}\,\bar{s}_{2,hkl}\,[\infty]$ TPa$^{-1}$ | $k_{2,hkl}$ |
|---|---|---|---|---|---|---|
| {100} | -6.49 | -6.49 | – | 28.76 | 28.76 | – |
| {110} | -6.49 | -6.49 | – | 28.76 | 28.76 | – |
| {002} | -5.36 | -5.77 | 0.43 | 25.82 | 26.88 | 0.44 |
| {101} | -6.83 | -6.73 | 0.35 | 29.54 | 29.54 | – |
| {102} | -6.69 | -6.69 | – | 29.49 | 29.49 | – |
| {110} | -6.49 | -6.49 | – | 28.76 | 28.76 | – |
| {103} | -6.37 | -6.45 | 0.54 | 28.81 | 28.81 | – |
| {200} | -6.49 | -6.49 | – | 28.76 | 28.76 | – |
| {112} | -6.87 | -6.75 | 0.36 | 29.94 | 29.62 | 0.36 |
| {201} | -6.58 | -6.58 | – | 29.06 | 29.06 | – |
| {004} | -5.36 | -5.77 | 0.43 | 25.82 | 26.88 | 0.44 |
| {202} | -6.83 | -6.73 | 0.35 | 29.54 | 29.54 | – |
| {104} | -6.07 | -6.25 | 0.47 | 27.84 | 28.25 | 0.52 |
| {203} | -6.87 | -6.76 | 0.36 | 30.00 | 29.68 | 0.36 |
| {120} | -6.49 | -6.49 | – | 28.76 | 28.76 | – |
| {211} | -6.55 | -6.55 | – | 28.95 | 28.95 | – |
| {114} | -6.62 | -6.62 | – | 29.29 | 29.29 | – |
| {105} | -5.87 | -6.12 | 0.45 | 27.28 | 27.87 | 0.48 |
| {122} | -6.74 | -6.67 | 0.33 | 29.33 | 29.33 | – |

Table 11: Surface and bulk X-ray elastic constants for silicon ($A$ = 0.11 and $a$ = 0.5431 nm). Single crystal elastic constants were taken from McSkimin & Andreatch Jr (1964)

| $\{hkl\}$ | $\bar{s}_{1,hkl}\,[0]$ TPa$^{-1}$ | $\bar{s}_{1,hkl}\,[\infty]$ TPa$^{-1}$ | $k_{1,hkl}$ | $\frac{1}{2}\,\bar{s}_{2,hkl}\,[0]$ TPa$^{-1}$ | $\frac{1}{2}\,\bar{s}_{2,hkl}\,[\infty]$ TPa$^{-1}$ | $k_{2,hkl}$ |
|---|---|---|---|---|---|---|
| {111} | -1.05 | -1.14 | 0.49 | 6.54 | 6.83 | 0.49 |
| {220} | -1.25 | -1.28 | 1.21 | 7.17 | 7.25 | 1.22 |
| {311} | -0.43 | -1.43 | -0.01 | 7.88 | 7.71 | -0.01 |
| {400} | -1.93 | -1.70 | 0.31 | 9.14 | 8.49 | 0.31 |
| {331} | -1.19 | -1.24 | 0.76 | 6.99 | 7.13 | 0.77 |
| {422} | -1.25 | -1.28 | 1.21 | 7.17 | 7.25 | 1.22 |
| {333} | -1.05 | -1.14 | 0.49 | 6.55 | 6.83 | 0.49 |
| {511} | -1.74 | -1.58 | 0.24 | 8.56 | 8.14 | 0.24 |
| {440} | -1.25 | -1.28 | 1.21 | 7.17 | 7.25 | 1.22 |
| {531} | -1.34 | -1.34 | – | 7.44 | 7.44 | – |
| {620} | -1.69 | -1.55 | 0.21 | 8.40 | 8.04 | 0.21 |

Table 12: Surface and bulk X-ray elastic constants for $\alpha-$zinc ($A = 0.64$, $a = 0.26649$ nm and $c = 0.49468$ nm). Single crystal elastic constants were taken from Alers & Neighbours (1908).

| $\{hkl\}$ | $\bar{s}_{1,hkl}\,[0]$ $TPa^{-1}$ | $\bar{s}_{1,hkl}\,[\infty]$ $TPa^{-1}$ | $k_{1,hkl}$ | $\frac{1}{2}\,\bar{s}_{2,hkl}\,[0]$ $TPa^{-1}$ | $\frac{1}{2}\,\bar{s}_{2,hkl}\,[\infty]$ $TPa^{-1}$ | $k_{2,hkl}$ |
|---|---|---|---|---|---|---|
| {100} | -2.47 | -2.42 | 0.73 | 10.90 | 10.90 | – |
| {110} | -2.47 | -2.42 | 0.73 | 10.90 | 10.90 | – |
| {002} | -5.51 | -3.90 | 0.34 | 23.14 | 18.50 | 0.37 |
| {101} | -2.07 | -2.28 | 0.10 | 10.68 | 11.05 | 0.16 |
| {102} | -2.42 | -2.42 | – | 12.35 | 12.35 | – |
| {110} | -2.47 | -2.42 | 0.73 | 10.90 | 10.90 | – |
| {103} | -3.10 | -2.77 | 0.42 | 15.15 | 14.01 | 0.44 |
| {200} | -2.47 | -2.42 | 0.73 | 10.90 | 10.90 | – |
| {112} | -2.05 | -2.28 | 0.11 | 10.76 | 11.17 | 0.15 |
| {201} | -2.36 | -2.36 | – | 10.83 | 10.89 | 0.05 |
| {004} | -5.51 | -3.90 | 0.34 | 23.14 | 18.50 | 0.37 |
| {202} | -2.07 | -2.28 | 0.10 | 10.68 | 11.05 | 0.16 |
| {104} | -3.75 | -3.07 | 0.37 | 17.40 | 15.29 | 0.40 |
| {203} | -2.01 | -2.30 | 0.07 | 11.20 | 11.57 | 0.03 |
| {120} | -2.47 | -2.42 | 0.73 | 10.90 | 10.90 | – |
| {211} | -2.38 | -2.38 | – | 10.89 | 10.89 | – |
| {114} | -2.60 | -2.52 | 0.89 | 13.22 | 12.88 | 0.69 |
| {105} | -4.21 | -3.29 | 0.35 | 18.95 | 16.16 | 0.38 |
| {122} | -2.22 | -2.31 | 0.13 | 10.69 | 10.93 | 0.15 |

Table 13: Surface and bulk X-ray elastic constants for $\alpha-$zirconium ($A = 0.05$, $a = 0.3232$ nm and $c = 0.5147$ nm). Single crystal elastic constants were taken from Fisher & Renken (1964).

| $\{hkl\}$ | $\bar{s}_{1,hkl}\,[0]$ $\mathrm{TPa}^{-1}$ | $\bar{s}_{1,hkl}\,[\infty]$ $\mathrm{TPa}^{-1}$ | $k_{1,hkl}$ | $\frac{1}{2}\,\bar{s}_{2,hkl}\,[0]$ $\mathrm{TPa}^{-1}$ | $\frac{1}{2}\,\bar{s}_{2,hkl}\,[\infty]$ $\mathrm{TPa}^{-1}$ | $k_{2,hkl}$ |
|---|---|---|---|---|---|---|
| {100} | -3.22 | -3.34 | 0.07 | 13.94 | 13.60 | -0.04 |
| {110} | -3.22 | -3.34 | 0.07 | 13.48 | 13.60 | -0.04 |
| {002} | -2.65 | -2.88 | 0.44 | 11.28 | 11.96 | 0.43 |
| {101} | -3.67 | -3.60 | 0.64 | 14.56 | 14.33 | 0.65 |
| {102} | -3.71 | -3.61 | 0.65 | 14.55 | 14.27 | 0.64 |
| {110} | -3.22 | -3.34 | 0.07 | 13.48 | 13.60 | -0.04 |
| {103} | -3.42 | -3.42 | – | 13.66 | 13.66 | – |
| {200} | -3.22 | -3.34 | 0.07 | 13.48 | 13.60 | -0.04 |
| {112} | -3.72 | -3.63 | 0.59 | 14.68 | 14.41 | 0.58 |
| {201} | -3.44 | -3.44 | – | 13.89 | 13.89 | – |
| {004} | -2.65 | -2.88 | 0.44 | 11.28 | 11.96 | 0.43 |
| {202} | -3.67 | -3.60 | 0.64 | 14.56 | 14.33 | 0.65 |
| {104} | -3.27 | -3.27 | – | 13.01 | 13.16 | 0.05 |
| {203} | -3.76 | -3.66 | 0.57 | 14.77 | 14.45 | 0.56 |
| {120} | -3.22 | -3.34 | 0.07 | 13.48 | 13.60 | -0.04 |
| {211} | -3.40 | -3.40 | – | 13.78 | 13.78 | – |
| {114} | -3.63 | -3.55 | 0.76 | 14.31 | 14.09 | 0.76 |
| {105} | -3.04 | -3.16 | 0.20 | 12.53 | 12.83 | 0.24 |
| {122} | -3.53 | -3.53 | – | 14.14 | 14.14 | – |

Table 14: Surface and bulk X-ray elastic constants for germanium ($A = 0.14$ and $a = 0.5658$ nm). Single crystal elastic constants were taken from Bogardus (1965).

| $\{hkl\}$ | $\bar{s}_{1,hkl}\,[0]$ $\mathrm{TPa}^{-1}$ | $\bar{s}_{1,hkl}\,[\infty]$ $\mathrm{TPa}^{-1}$ | $k_{1,hkl}$ | $\frac{1}{2}\,\bar{s}_{2,hkl}\,[0]$ $\mathrm{TPa}^{-1}$ | $\frac{1}{2}\,\bar{s}_{2,hkl}\,[\infty]$ $\mathrm{TPa}^{-1}$ | $k_{2,hkl}$ |
|---|---|---|---|---|---|---|
| {111} | -1.16 | -1.27 | 0.41 | 7.92 | 8.25 | 0.41 |
| {220} | -1.46 | -1.46 | – | 8.81 | 8.81 | – |
| {311} | -1.78 | -1.67 | 0.44 | 9.78 | 9.45 | 0.45 |
| {400} | -2.34 | -2.04 | 0.44 | 11.45 | 10.56 | 0.43 |
| {331} | -1.37 | -1.40 | 0.36 | 8.54 | 8.65 | 0.36 |
| {422} | -1.46 | -1.46 | – | 8.81 | 8.81 | – |
| {333} | -1.16 | -1.27 | 0.41 | 7.92 | 8.25 | 0.41 |
| {511} | -2.09 | -1.87 | 0.44 | 10.70 | 10.06 | 0.44 |
| {440} | -1.46 | -1.46 | – | 8.81 | 8.81 | – |
| {531} | -1.59 | -1.54 | 0.48 | 9.21 | 9.08 | 0.47 |
| {620} | -2.02 | -1.82 | 0.44 | 10.49 | 9.92 | 0.44 |

Table 15: Surface and bulk X-ray elastic constants for cadmium telluride ($A = 0.38$ and $a = 0.648$ nm). Single crystal elastic constants were taken from Deligoz *et al.* (2006).

| $\{hkl\}$ | $\bar{s}_{1,hkl}\,[0]$ TPa$^{-1}$ | $\bar{s}_{1,hkl}\,[\infty]$ TPa$^{-1}$ | $k_{1,hkl}$ | $\frac{1}{2}\,\bar{s}_{2,hkl}\,[0]$ TPa$^{-1}$ | $\frac{1}{2}\,\bar{s}_{2,hkl}\,[\infty]$ TPa$^{-1}$ | $k_{2,hkl}$ |
|---|---|---|---|---|---|---|
| {111} | -6.48 | -7.22 | 0.43 | 27.35 | 29.56 | 0.43 |
| {200} | -14.06 | -11.76 | 0.37 | 49.83 | 43.18 | 0.37 |
| {220} | -8.24 | -8.35 | 0.84 | 32.74 | 32.96 | 0.84 |
| {311} | -10.27 | -9.61 | 0.35 | 38.84 | 36.75 | 0.35 |
| {222} | -6.48 | -7.22 | 0.43 | 27.47 | 29.56 | 0.43 |
| {400} | -14.06 | -11.76 | 0.37 | 49.83 | 43.18 | 0.37 |
| {420} | -10.21 | -9.57 | 0.35 | 38.64 | 36.62 | 0.35 |
| {422} | -8.24 | -8.35 | 0.84 | 32.74 | 32.96 | 0.84 |
| {440} | -8.24 | -8.35 | 0.84 | 32.74 | 32.96 | 0.84 |
| {600} | -14.06 | -11.76 | 0.37 | 49.83 | 43.18 | 0.37 |
| {442} | -7.25 | -7.72 | 0.47 | 29.79 | 31.07 | 0.47 |

Table 16: Surface and bulk X-ray elastic constants for titanium carbide ($A = 0.01$ and $a = 0.43186$ nm). Single crystal elastic constants were taken from Gilman & Roberts (1961).

| $\{hkl\}$ | $\bar{s}_{1,hkl}\,[0]$ TPa$^{-1}$ | $\bar{s}_{1,hkl}\,[\infty]$ TPa$^{-1}$ | $k_{1,hkl}$ | $\frac{1}{2}\,\bar{s}_{2,hkl}\,[0]$ TPa$^{-1}$ | $\frac{1}{2}\,\bar{s}_{2,hkl}\,[\infty]$ TPa$^{-1}$ | $k_{2,hkl}$ |
|---|---|---|---|---|---|---|
| {111} | -0.48 | -0.47 | 0.48 | 2.80 | 2.80 | – |
| {200} | -0.42 | -0.43 | 0.49 | 2.62 | 2.66 | 0.49 |
| {220} | -0.46 | -0.46 | – | 2.77 | 2.77 | – |
| {311} | -0.45 | -0.45 | – | 2.73 | 2.73 | – |
| {222} | -0.48 | -0.47 | 0.48 | 2.80 | 2.80 | – |
| {400} | -0.42 | -0.43 | 0.49 | 2.62 | 2.66 | 0.49 |
| {331} | -0.47 | -0.47 | – | 2.78 | 2.78 | – |
| {420} | -0.45 | -0.45 | – | 2.73 | 2.73 | – |
| {422} | -0.46 | -0.46 | – | 2.77 | 2.77 | – |
| {333} | -0.48 | -0.47 | 0.48 | 2.80 | 2.80 | – |
| {511} | -0.43 | -0.44 | 0.47 | 2.69 | 2.69 | – |

Table 17: Surface and bulk X-ray elastic constants for 2H silicon carbide ($A = 0.08$, $a = 0.30763$ nm and $c = 0.50486$ nm). Single crystal elastic constants were taken from Pizzagalli (2021).

| $\{hkl\}$ | $\bar{s}_{1,hkl}\,[0]$ $\mathrm{TPa}^{-1}$ | $\bar{s}_{1,hkl}\,[\infty]$ $\mathrm{TPa}^{-1}$ | $k_{1,hkl}$ | $\frac{1}{2}\,\bar{s}_{2,hkl}\,[0]$ $\mathrm{TPa}^{-1}$ | $\frac{1}{2}\,\bar{s}_{2,hkl}\,[\infty]$ $\mathrm{TPa}^{-1}$ | $k_{2,hkl}$ |
|---|---|---|---|---|---|---|
| {100} | -0.27 | -0.29 | 0.44 | 2.32 | 2.40 | 0.48 |
| {002} | -0.20 | -0.24 | 0.40 | 2.10 | 2.22 | 0.39 |
| {101} | -0.39 | -0.38 | 0.35 | 2.69 | 2.64 | 0.33 |
| {102} | -0.44 | -0.40 | 0.33 | 2.82 | 2.72 | 0.35 |
| {2-10} | -0.27 | -0.29 | 0.44 | 2.32 | 2.40 | 0.48 |
| {103} | -0.39 | -0.37 | 0.21 | 2.66 | 2.61 | 0.27 |
| {2-12} | -0.41 | -0.39 | 0.36 | 2.75 | 2.68 | 0.35 |
| {201} | -0.31 | -0.32 | 0.49 | 2.46 | 2.49 | 0.59 |
| {203} | -0.44 | -0.40 | 0.36 | 2.82 | 2.73 | 0.36 |
| {210} | -0.27 | -0.29 | 0.44 | 2.32 | 2.40 | 0.48 |
| {211} | -0.29 | -0.31 | 0.47 | 2.41 | 2.45 | 0.53 |
| {114} | -0.43 | -0.39 | 0.30 | 2.78 | 2.70 | 0.34 |
| {105} | -0.30 | -0.30 | 0.92 | 2.39 | 2.43 | 0.62 |
| {212} | -0.35 | -0.35 | – | 2.58 | 2.57 | 0.04 |
| {300} | -0.27 | -0.29 | 0.44 | 2.32 | 2.40 | 0.48 |
| {213} | -0.41 | -0.39 | 0.36 | 2.74 | 2.68 | 0.35 |
| {214} | -0.44 | -0.40 | 0.36 | 2.82 | 2.73 | 0.36 |

Table 18: Surface and bulk X-ray elastic constants for 4H silicon carbide ($A = 0.06$, $a = 0.30784$ nm and $c = 1.00776$ nm). Single crystal elastic constants were taken from Pizzagalli (2021).

| $\{hkl\}$ | $\bar{s}_{1,hkl}$ [0] TPa$^{-1}$ | $\bar{s}_{1,hkl}$ [$\infty$] TPa$^{-1}$ | $k_{1,hkl}$ | $\frac{1}{2}$ $\bar{s}_{2,hkl}$ [0] TPa$^{-1}$ | $\frac{1}{2}$ $\bar{s}_{2,hkl}$ [$\infty$] TPa$^{-1}$ | $k_{2,hkl}$ |
|---|---|---|---|---|---|---|
| {102} | -0.38 | -0.37 | 0.42 | 2.67 | 2.62 | 0.41 |
| {103} | -0.42 | -0.39 | 0.38 | 2.77 | 2.69 | 0.39 |
| {104} | -0.42 | -0.39 | 0.33 | 2.77 | 2.68 | 0.34 |
| {105} | -0.41 | -0.37 | 0.27 | 2.71 | 2.63 | 0.27 |
| {110} | -0.28 | -0.30 | 0.41 | 2.37 | 2.41 | 0.42 |
| {106} | -0.39 | -0.35 | 0.20 | 2.63 | 2.57 | 0.19 |
| {202} | -0.32 | -0.32 | 0.40 | 2.49 | 2.49 | – |
| {205} | -0.41 | -0.38 | 0.40 | 2.73 | 2.66 | 0.40 |
| {206} | -0.42 | -0.39 | 0.38 | 2.77 | 2.69 | 0.39 |
| {109} | -0.30 | -0.31 | -0.10 | 2.43 | 2.43 | – |
| {212} | -0.30 | -0.31 | 0.41 | 2.46 | 2.46 | – |
| {213} | -0.33 | -0.33 | – | 2.51 | 2.51 | – |
| {1010} | -0.30 | -0.30 | – | 2.40 | 2.40 | – |
| {214} | -0.35 | -0.35 | 0.43 | 2.58 | 2.56 | 0.39 |
| {215} | -0.38 | -0.36 | 0.42 | 2.65 | 2.61 | 0.40 |
| {300} | -0.28 | -0.30 | 0.41 | 2.37 | 2.41 | 0.42 |
| {209} | -0.42 | -0.38 | 0.30 | 2.74 | 2.65 | 0.31 |

Table 19: Surface and bulk X-ray elastic constants for 6H silicon carbide ($A = 0.05$, $a = 0.30793$ nm and $c = 1.51091$ nm). Single crystal elastic constants were taken from Pizzagalli (2021).

| $\{hkl\}$ | $\bar{s}_{1,hkl}\,[0]$ TPa$^{-1}$ | $\bar{s}_{1,hkl}\,[\infty]$ TPa$^{-1}$ | $k_{1,hkl}$ | $\frac{1}{2}\,\bar{s}_{2,hkl}\,[0]$ TPa$^{-1}$ | $\frac{1}{2}\,\bar{s}_{2,hkl}\,[\infty]$ TPa$^{-1}$ | $k_{2,hkl}$ |
|---|---|---|---|---|---|---|
| {101} | -0.30 | -0.31 | 0.24 | 2.42 | 2.46 | 0.29 |
| {006} | -0.20 | -0.24 | 0.40 | 2.11 | 2.22 | 0.37 |
| {102} | -0.34 | -0.34 | – | 2.54 | 2.54 | – |
| {103} | -0.38 | -0.36 | 0.58 | 2.66 | 2.62 | 0.53 |
| {104} | -0.40 | -0.38 | 0.46 | 2.73 | 2.66 | 0.45 |
| {107} | -0.39 | -0.37 | 0.36 | 2.69 | 2.63 | 0.44 |
| {108} | -0.38 | -0.36 | 0.34 | 2.64 | 2.60 | 0.45 |
| {2-10} | -0.28 | -0.30 | 0.30 | 2.38 | 2.43 | 0.35 |
| {109} | -0.36 | -0.35 | 0.30 | 2.59 | 2.56 | 0.49 |
| {2-16} | -0.39 | -0.37 | 0.50 | 2.70 | 2.64 | 0.49 |
| {208} | -0.40 | -0.38 | 0.46 | 2.73 | 2.66 | 0.45 |
| {209} | -0.41 | -0.38 | 0.43 | 2.74 | 2.67 | 0.44 |
| {211} | -0.28 | -0.30 | 0.29 | 2.38 | 2.43 | 0.34 |
| {213} | -0.30 | -0.31 | 0.21 | 2.44 | 2.47 | 0.28 |
| {1015} | -0.29 | -0.30 | 0.50 | 2.36 | 2.40 | 0.33 |
| {217} | -0.36 | -0.36 | 0.73 | 2.62 | 2.59 | 0.60 |
| {219} | -0.39 | -0.37 | 0.51 | 2.69 | 2.64 | 0.49 |

Table 20: Surface and bulk X-ray elastic constants for 3C silicon carbide ($A = 0.18$ and $a = 0.4348$ nm). Single crystal elastic constants were taken from Pizzagalli (2021).

| $\{hkl\}$ | $\bar{s}_{1,hkl}\,[0]$ TPa$^{-1}$ | $\bar{s}_{1,hkl}\,[\infty]$ TPa$^{-1}$ | $k_{1,hkl}$ | $\frac{1}{2}\,\bar{s}_{2,hkl}\,[0]$ TPa$^{-1}$ | $\frac{1}{2}\,\bar{s}_{2,hkl}\,[\infty]$ TPa$^{-1}$ | $k_{2,hkl}$ |
|---|---|---|---|---|---|---|
| {111} | -0.18 | -0.22 | 0.34 | 2.10 | 2.22 | 0.34 |
| {200} | -0.56 | -0.47 | 0.36 | 3.24 | 2.96 | 0.36 |
| {220} | -0.27 | -0.28 | 0.11 | 2.38 | 2.41 | 0.11 |
| {311} | -0.38 | -0.35 | 0.47 | 2.70 | 2.61 | 0.47 |
| {222} | -0.18 | -0.22 | 0.34 | 2.11 | 2.22 | 0.34 |
| {400} | -0.56 | -0.47 | 0.36 | 3.24 | 2.96 | 0.36 |
| {331} | -0.24 | -0.26 | 0.24 | 2.30 | 2.35 | 0.24 |
| {420} | -0.37 | -0.35 | 0.48 | 2.69 | 2.61 | 0.47 |
| {422} | -0.27 | -0.28 | 0.11 | 2.38 | 2.41 | 0.11 |
| {333} | -0.18 | -0.22 | 0.34 | 2.11 | 2.22 | 0.34 |
| {511} | -0.48 | -0.42 | 0.39 | 3.00 | 2.81 | 0.39 |

Table 21: Surface and bulk X-ray elastic constants for tungsten carbide ($A = 0.07$, $a = 0.29059$ nm and $c = 0.28367$ nm). Single crystal elastic constants were taken from Lee & Gilmore (1982).

| $\{hkl\}$ | $\bar{s}_{1,hkl}$ [0] $\mathrm{TPa}^{-1}$ | $\bar{s}_{1,hkl}$ [$\infty$] $\mathrm{TPa}^{-1}$ | $k_{1,hkl}$ | $\frac{1}{2}\bar{s}_{2,hkl}$ [0] $\mathrm{TPa}^{-1}$ | $\frac{1}{2}\bar{s}_{2,hkl}$ [$\infty$] $\mathrm{TPa}^{-1}$ | $k_{2,hkl}$ |
|---|---|---|---|---|---|---|
| {001} | -0.32 | -0.33 | 0.35 | 1.60 | 1.64 | 0.38 |
| {100} | -0.38 | -0.36 | 0.46 | 1.96 | 1.91 | 0.44 |
| {101} | -0.31 | -0.32 | 0.19 | 1.68 | 1.70 | 0.21 |
| {110} | -0.38 | -0.36 | 0.46 | 1.96 | 1.91 | 0.44 |
| {002} | -0.32 | -0.33 | 0.35 | 1.61 | 1.64 | 0.38 |
| {111} | -0.34 | -0.34 | – | 1.79 | 1.79 | – |
| {200} | -0.38 | -0.36 | 0.46 | 1.96 | 1.91 | 0.44 |
| {102} | -0.31 | -0.32 | 0.38 | 1.60 | 1.64 | 0.39 |
| {201} | -0.35 | -0.34 | 1.17 | 1.82 | 1.82 | – |
| {112} | -0.31 | -0.32 | 0.28 | 1.65 | 1.68 | 0.30 |
| {210} | -0.38 | -0.36 | 0.46 | 1.96 | 1.91 | 0.44 |
| {003} | -0.32 | -0.33 | 0.35 | 1.61 | 1.64 | 0.38 |
| {202} | -0.31 | -0.32 | 0.19 | 1.68 | 1.70 | 0.21 |
| {211} | -0.36 | -0.35 | 0.72 | 1.88 | 1.85 | 0.63 |
| {103} | -0.31 | -0.32 | 0.38 | 1.60 | 1.64 | 0.38 |
| {300} | -0.38 | -0.36 | 0.46 | 1.96 | 1.91 | 0.44 |
| {301} | -0.36 | -0.35 | 0.64 | 1.89 | 1.86 | 0.58 |

Table 22: Surface and bulk X-ray elastic constants for silicon nitride ($A = 0.14$, $a = 0.76044$ nm and $c = 0.29075$ nm). Single crystal elastic constants were taken from Vogelgesang *et al.* (2000).

| $\{hkl\}$ | $\bar{s}_{1,hkl}\,[0]$ $\mathrm{TPa}^{-1}$ | $\bar{s}_{1,hkl}\,[\infty]$ $\mathrm{TPa}^{-1}$ | $k_{1,hkl}$ | $\frac{1}{2}\,\bar{s}_{2,hkl}\,[0]$ $\mathrm{TPa}^{-1}$ | $\frac{1}{2}\,\bar{s}_{2,hkl}\,[\infty]$ $\mathrm{TPa}^{-1}$ | $k_{2,hkl}$ |
|---|---|---|---|---|---|---|
| {100} | -0.82 | -0.82 | – | 3.80 | 3.80 | – |
| {110} | -0.82 | -0.82 | – | 3.80 | 3.80 | – |
| {200} | -0.82 | -0.82 | – | 3.80 | 3.80 | – |
| {101} | -0.73 | -0.75 | 1.58 | 3.40 | 3.48 | 1.23 |
| {210} | -0.82 | -0.82 | – | 3.80 | 3.80 | – |
| {301} | -1.01 | -0.96 | 0.36 | 4.32 | 4.16 | 0.38 |
| {221} | -1.01 | -0.95 | 0.40 | 4.30 | 4.15 | 0.40 |
| {320} | -0.82 | -0.82 | – | 3.80 | 3.80 | – |
| {002} | -0.48 | -0.58 | 0.56 | 2.64 | 2.93 | 0.54 |
| {231} | -0.98 | -0.93 | 0.45 | 4.22 | 4.10 | 0.41 |
| {411} | -0.97 | -0.93 | 0.46 | 4.20 | 4.09 | 0.42 |
| {330} | -0.82 | -0.82 | – | 3.80 | 3.80 | – |
| {212} | -0.83 | -0.83 | – | 3.71 | 3.71 | – |
| {511} | -0.94 | -0.91 | 0.49 | 4.11 | 4.03 | 0.41 |
| {430} | -0.82 | -0.82 | – | 3.80 | 3.80 | – |
| {232} | -0.99 | -0.94 | 0.21 | 4.22 | 4.08 | 0.30 |
| {142} | -1.00 | -0.95 | 0.24 | 4.25 | 4.10 | 0.32 |
| {610} | -0.82 | -0.82 | – | 3.80 | 3.80 | – |
| {521} | -0.92 | -0.89 | 0.51 | 4.06 | 4.00 | 0.40 |

Table 23: Surface and bulk X-ray elastic constants for titanium nitride ($A = 0.06$ and $a = 0.424$ nm). Single crystal elastic constants were taken from Fodil *et al.* (2014).

| $\{hkl\}$ | $\bar{s}_{1,hkl}\,[0]$ $\mathrm{TPa}^{-1}$ | $\bar{s}_{1,hkl}\,[\infty]$ $\mathrm{TPa}^{-1}$ | $k_{1,hkl}$ | $\frac{1}{2}\,\bar{s}_{2,hkl}\,[0]$ $\mathrm{TPa}^{-1}$ | $\frac{1}{2}\,\bar{s}_{2,hkl}\,[\infty]$ $\mathrm{TPa}^{-1}$ | $k_{2,hkl}$ |
|---|---|---|---|---|---|---|
| {111} | -0.64 | -0.60 | 0.43 | 2.95 | 2.85 | 0.43 |
| {200} | -0.41 | -0.45 | 0.40 | 2.29 | 2.40 | 0.40 |
| {220} | -0.58 | -0.56 | 0.57 | 2.78 | 2.74 | 0.58 |
| {311} | -0.51 | -0.52 | 0.14 | 2.59 | 2.61 | 0.15 |
| {222} | -0.64 | -0.60 | 0.43 | 2.95 | 2.85 | 0.43 |
| {400} | -0.41 | -0.45 | 0.40 | 2.29 | 2.40 | 0.40 |
| {331} | -0.59 | -0.58 | 0.50 | 2.83 | 2.77 | 0.51 |
| {420} | -0.51 | -0.52 | 0.13 | 2.60 | 2.62 | 0.13 |
| {422} | -0.58 | -0.56 | 0.57 | 2.78 | 2.74 | 0.58 |
| {333} | -0.64 | -0.60 | 0.43 | 2.95 | 2.85 | 0.43 |
| {511} | -0.46 | -0.48 | 0.34 | 2.42 | 2.50 | 0.34 |

Table 24: Surface and bulk X-ray elastic constants for $\alpha$-corundum ($A = 0.07$, $a = 0.47606$ nm and $c = 1.2994$ nm). Single crystal elastic constants were taken from Tefft (1966).

| $\{hkl\}$ | $\bar{s}_{1,hkl}\,[0]$ TPa$^{-1}$ | $\bar{s}_{1,hkl}\,[\infty]$ TPa$^{-1}$ | $k_{1,hkl}$ | $\frac{1}{2}\,\bar{s}_{2,hkl}\,[0]$ TPa$^{-1}$ | $\frac{1}{2}\,\bar{s}_{2,hkl}\,[\infty]$ TPa$^{-1}$ | $k_{2,hkl}$ |
|---|---|---|---|---|---|---|
| {012} | -0.51 | -0.54 | 0.13 | 2.85 | 2.93 | 0.13 |
| {104} | -0.67 | -0.64 | 0.62 | 3.37 | 3.26 | 0.62 |
| {110} | -0.55 | -0.55 | – | 2.95 | 2.95 | – |
| {113} | -0.62 | -0.61 | 0.96 | 3.19 | 3.13 | 0.97 |
| {024} | -0.51 | -0.54 | 0.13 | 2.88 | 2.93 | 0.13 |
| {116} | -0.62 | -0.61 | 1.01 | 3.22 | 3.16 | 0.95 |
| {214} | -0.67 | -0.64 | 0.65 | 3.33 | 3.22 | 0.65 |
| {300} | -0.55 | -0.55 | – | 2.95 | 2.95 | – |
| {226} | -0.62 | -0.61 | 0.96 | 3.19 | 3.13 | 0.97 |
| {2110} | -0.65 | -0.62 | 0.72 | 3.31 | 3.22 | 0.70 |
| {1310} | -0.57 | -0.57 | – | 3.04 | 3.04 | – |
| {3012} | -0.67 | -0.64 | 0.62 | 3.37 | 3.26 | 0.62 |
| {2014} | -0.53 | -0.55 | 0.16 | 2.99 | 3.02 | 0.06 |
| {416} | -0.69 | -0.65 | 0.60 | 3.38 | 3.25 | 0.60 |
| {4010} | -0.74 | -0.68 | 0.45 | 3.57 | 3.38 | 0.45 |
| {330} | -0.55 | -0.55 | – | 2.95 | 2.95 | – |

Table 25: Surface and bulk X-ray elastic constants for cubic zirconia ($A = 0.52$ and $a = 0.509$ nm). Single crystal elastic constants were taken from Kandil *et al.* (1984).

| $\{hkl\}$ | $\bar{s}_{1,hkl}\,[0]$ TPa$^{-1}$ | $\bar{s}_{1,hkl}\,[\infty]$ TPa$^{-1}$ | $k_{1,hkl}$ | $\frac{1}{2}\,\bar{s}_{2,hkl}\,[0]$ TPa$^{-1}$ | $\frac{1}{2}\,\bar{s}_{2,hkl}\,[\infty]$ TPa$^{-1}$ | $k_{2,hkl}$ |
|---|---|---|---|---|---|---|
| {111} | -2.10 | -1.79 | 0.39 | 8.00 | 7.06 | 0.39 |
| {200} | -0.71 | -0.87 | 0.50 | 3.82 | 4.28 | 0.50 |
| {220} | -1.77 | -1.56 | 0.30 | 6.90 | 6.35 | 0.30 |
| {311} | -1.47 | -1.30 | 0.06 | 5.76 | 5.57 | 0.05 |
| {222} | -2.10 | -1.79 | 0.39 | 7.94 | 7.06 | 0.39 |
| {400} | -0.71 | -0.87 | 0.50 | 3.82 | 4.28 | 0.50 |
| {331} | -1.86 | -1.62 | 0.33 | 7.20 | 6.56 | 0.33 |
| {420} | -1.46 | -1.30 | 0.07 | 5.80 | 5.60 | 0.07 |
| {422} | -1.77 | -1.56 | 0.30 | 6.90 | 6.35 | 0.30 |
| {333} | -2.10 | -1.79 | 0.39 | 7.94 | 7.06 | 0.39 |
| {511} | -1.00 | -1.06 | 0.94 | 4.69 | 4.85 | 0.93 |
| {440} | -1.77 | -1.56 | 0.30 | 6.90 | 6.35 | 0.30 |
| {531} | -1.61 | -1.45 | 0.24 | 6.43 | 6.03 | 0.24 |
| {442} | -1.95 | -1.69 | 0.35 | 7.48 | 6.75 | 0.35 |
| {600} | -0.71 | -0.87 | 0.50 | 3.82 | 4.28 | 0.50 |
| {620} | -1.09 | -1.11 | 2.11 | 4.94 | 5.02 | 2.14 |
| {533} | -1.92 | -1.66 | 0.34 | 7.36 | 6.67 | 0.34 |

Table 26: Surface and bulk X-ray elastic constants for tetragonal zirconia ($A = 0.23$, $a = 0.3579$ nm and $c = 0.5165$ nm). Single crystal elastic constants were taken from Kisi & Howard (1998).

| $\{hkl\}$ | $\bar{s}_{1,hkl}\,[0]$ $\mathrm{TPa}^{-1}$ | $\bar{s}_{1,hkl}\,[\infty]$ $\mathrm{TPa}^{-1}$ | $k_{1,hkl}$ | $\frac{1}{2}\,\bar{s}_{2,hkl}\,[0]$ $\mathrm{TPa}^{-1}$ | $\frac{1}{2}\,\bar{s}_{2,hkl}\,[\infty]$ $\mathrm{TPa}^{-1}$ | $k_{2,hkl}$ |
|---|---|---|---|---|---|---|
| {101} | -1.53 | -1.45 | 0.29 | 6.81 | 6.55 | 0.31 |
| {002} | -0.74 | -0.93 | 0.41 | 4.91 | 5.39 | 0.39 |
| {110} | -1.57 | -1.47 | 0.23 | 6.67 | 6.44 | 0.21 |
| {112} | -1.77 | -1.61 | 0.39 | 7.62 | 7.14 | 0.39 |
| {200} | -0.92 | -1.02 | 0.61 | 4.75 | 5.11 | 0.59 |
| {103} | -1.26 | -1.26 | – | 6.28 | 6.28 | – |
| {211} | -1.49 | -1.42 | 0.23 | 6.53 | 6.33 | 0.21 |
| {202} | -1.53 | -1.45 | 0.29 | 6.81 | 6.55 | 0.31 |
| {004} | -0.74 | -0.93 | 0.41 | 4.91 | 5.39 | 0.39 |
| {220} | -1.57 | -1.47 | 0.23 | 6.67 | 6.44 | 0.21 |
| {213} | -1.71 | -1.58 | 0.41 | 7.45 | 7.02 | 0.41 |
| {301} | -1.06 | -1.12 | 0.86 | 5.21 | 5.42 | 0.77 |
| {204} | -1.51 | -1.43 | 0.24 | 6.93 | 6.69 | 0.28 |
| {312} | -1.47 | -1.40 | 0.21 | 6.51 | 6.33 | 0.20 |
| {105} | -0.99 | -1.09 | 0.55 | 5.58 | 5.81 | 0.58 |
| {224} | -1.77 | -1.61 | 0.39 | 7.62 | 7.14 | 0.39 |
| {400} | -0.92 | -1.02 | 0.61 | 4.75 | 5.11 | 0.59 |
| {215} | -1.49 | -1.41 | 0.19 | 6.89 | 6.66 | 0.25 |
| {314} | -1.66 | -1.54 | 0.41 | 7.29 | 6.91 | 0.41 |
